# A Conversational Agent Using Natural Language Processing for Postpartum Care for New Mothers: Development and Engagement Analysis

**DOI:** 10.2196/58454

**Published:** 2025-04-22

**Authors:** Kirstin Leitner, Clare Cutri-French, Abigail Mandel, Lori Christ, Nathaneal Koelper, Meaghan McCabe, Emily Seltzer, Laura Scalise, James A Colbert, Anuja Dokras, Roy Rosin, Lisa Levine

**Affiliations:** 1 Department of Obstetrics and Gynecology, University of Pennsylvania, 3701 Market Street, 3rd Floor, Philadelphia, PA, 19104, United States, 1 651-492-3856; 2 Hospital of the University of Pennsylvania, Philadelphia, PA, United States; 3 Medical University of South Carolina, Charleston, SC, United States; 4 Intensive Care Nursery, Department of Pediatrics, University of Pennsylvania, Philadelphia, PA, United States; 5 School of Medicine, University of Pennsylvania, Philadelphia, PA, United States; 6 Maternal Fetal Medicine Research Center, School of Medicine, University of Pennsylvania, Philadelphia, PA, United States; 7 Penn Medicine Center for Health Care Transformation and Innovation, Philadelphia, PA, United States; 8 Memora Health, San Francisco, CA, United States; 9 Division of Reproductive Endocrinology and Infertility, Department of Obstetrics and Gynecology, University of Pennsylvania, Philadelphia, PA, United States; 10 Penn Medicine, Philadelphia, PA, United States; 11 Division of Maternal Fetal Medicine, Department of Obstetrics and Gynecology, University of Pennsylvania, Philadelphia, PA, United States

**Keywords:** conversational agent, postpartum care, text messaging, postpartum, natural language processing, pregnancy, parents, newborns, development, patient engagement, physical recovery, infant, infant care, survey, breastfeeding, support, patient support, patient satisfaction

## Abstract

**Background:**

The “fourth trimester,” or postpartum time period, remains a critical phase of pregnancy that significantly impacts parents and newborns. Care poses challenges due to complex individual needs as well as low attendance rates at routine appointments. A comprehensive technological solution could provide a holistic and equitable solution to meet care goals.

**Objective:**

This paper describes the development of patient engagement data with a novel postpartum conversational agent that uses natural language processing to support patients post partum.

**Methods:**

We report on the development of a postpartum conversational agent from concept to usable product as well as the patient engagement with this technology. Content for the program was developed using patient- and provider-based input and clinical algorithms. Our program offered 2-way communication to patients and details on physical recovery, lactation support, infant care, and warning signs for problems. This was iterated upon by our core clinical team and an external expert clinical panel before being tested on patients. Patients eligible for discharge around 24 hours after delivery who had delivered a singleton full-term infant vaginally were offered use of the program. Patient demographics, accuracy, and patient engagement were collected over the first 6 months of use.

**Results:**

A total of 290 patients used our conversational agent over the first 6 months, of which 112 (38.6%) were first time parents and 162 (56%) were Black. In total, 286 (98.6%) patients interacted with the platform at least once, 271 patients (93.4%) completed at least one survey, and 151 (52%) patients asked a question. First time parents and those breastfeeding their infants had higher rates of engagement overall. Black patients were more likely to promote the program than White patients (*P*=.047). The overall accuracy of the conversational agent during the first 6 months was 77%.

**Conclusions:**

It is possible to develop a comprehensive, automated postpartum conversational agent. The use of such a technology to support patients postdischarge appears to be acceptable with very high engagement and patient satisfaction.

## Introduction

The “fourth trimester,” or postpartum time period, is often a forgotten “trimester” of pregnancy, yet plays a critical role in parental and newborn well-being. While undergoing numerous physiologic and emotional changes following birth, patients are also susceptible to complications such as infection, thrombosis, and hypertensive disorders as well as the new onset or exacerbation of mental health disorders [1,2]. The potential for medical complications post partum is of particular concern as over one-half of pregnancy related deaths occur after the birth of the infant [3,4]. These deaths also disproportionately affect Black women with maternal mortality rates nearly 3-times that of non-Hispanic White women [5]. The American College of Obstetricians and Gynecologists (ACOG) recommends that care during the postpartum period should be an “ongoing process” rather than the traditional 1-time postpartum visit [6]. A study evaluating the clinical features of postpartum presentation for emergency care indicated that while rates are overall low around 5%, most visits occur within the first 2 weeks post partum and are more likely to occur in Black patients [7]. Yet, even when follow up is recommended, nearly 50% of patients in the United States do not attend their routine postpartum appointment and adding additional clinic visits to increase access is impractical and impossible for both patients and clinicians [8].

This gap between patient needs, clinical recommendations and reality of health care access presents a significant challenge to patients and practicing providers. Innovative methods of identifying needs and providing ongoing care for the postpartum patient are needed without added burden to already overextended providers. A wide range of SMS text messaging health care interventions have been developed and trialed with varied success [9]. Within the realm of postpartum care these innovations have largely focused on specific individual conditions regarding postpartum recovery such as breastfeeding [10-12], blood pressure monitoring [13], and weight loss [14-17]. While many of these interventions have shown great promise in improving compliance with care and reducing health care disparities [13], there are limited comprehensive technologic interventions to support patients holistically during the fourth trimester. A technology-based solution has the potential to meet ACOG’s goals of continued contact and comprehensive postpartum care for patients. In this manuscript we describe the development of a novel comprehensive postpartum conversational agent, which uses natural language processing (NLP) to provide anticipatory guidance and respond to patients’ questions in real time. We also describe patient engagement and satisfaction with this novel technology.

## Methods

### Program Design and Content Development

We sought to create a comprehensive technology-based postpartum support program, “Healing at Home,” which would provide 24/7 support to individuals through the use of SMS text messages for 6 weeks post partum. Content included anticipatory guidance regarding physical recovery, infant care and feeding, clinical algorithms to respond to urgent needs and postpartum depression screening through the Edinburgh Postnatal Depression Screen (EPDS). The EPDS is a clinically validated 10-question survey that is considered the standard for screening patients for postpartum depression. We postulated that a 24/7 SMS text message—based holistic support would result in increased engagement of patients and allow providers to identify symptoms before they resulted in complications. Automation of messaging and responses, alongside the ability to focus attention efficiently on patients with demonstrated higher needs, could also minimize care team workload. Patients could be quickly connected to their care team and receive in-the-moment answers to their concerns.

We used a multipronged approach to optimize discharge planning and maintain postpartum connection for patients delivering at the Hospital of the University of Pennsylvania (HUP), described in detail by Gaulton et al [18]. We called this program of optimized discharge planning and increased postpartum support “Healing at Home.” Pertinent to the innovation described here, this preintervention pilot leveraged a “fake back end” SMS text message—based support during business hours (8 AM-5 PM) for patients for the first 6 weeks post partum. During this preintervention phase described by Gaulton et al [18], 90 patients were enrolled and encouraged to text their questions to the team. Text messages were monitored by nonclinical as well as clinical staff viewing and responding to patients. The team used a clinical reference guide, which was elaborated on throughout the pilot, outlining responses to frequently asked questions. While this method was effective at connecting with patients, it required significant time monitoring messages and responding to patients. Over 2000 text messages were exchanged with this cohort of 90 patients. In addition, we identified highly complex and individual needs ranging from inquiries about physical recovery specific to delivery mode (vaginal vs cesarean) to care of newborns (diapering and umbilical cord care) and infant feeding difficulties (pain with breastfeeding, difficulty pumping, and preparing formula). This complexity led us to conclude that a “simple” algorithmic approach was unlikely to be successful in providing this population with the holistic support required.

Conversational agents are designed to simulate conversation with human users and have become nearly ubiquitous in business, but their development within health care has been slow. Given the complexity and individualized needs of the postpartum patient we postulated that a conversational agent using NLP might be a good solution and be acceptable to this population. We envisioned a 24/7 available SMS text message—based support program that interpreted patients’ postpartum concerns, responded in real sentences and could also alert clinicians in real time when appropriate.

### Conversational Agent Development

**Figure 1.**
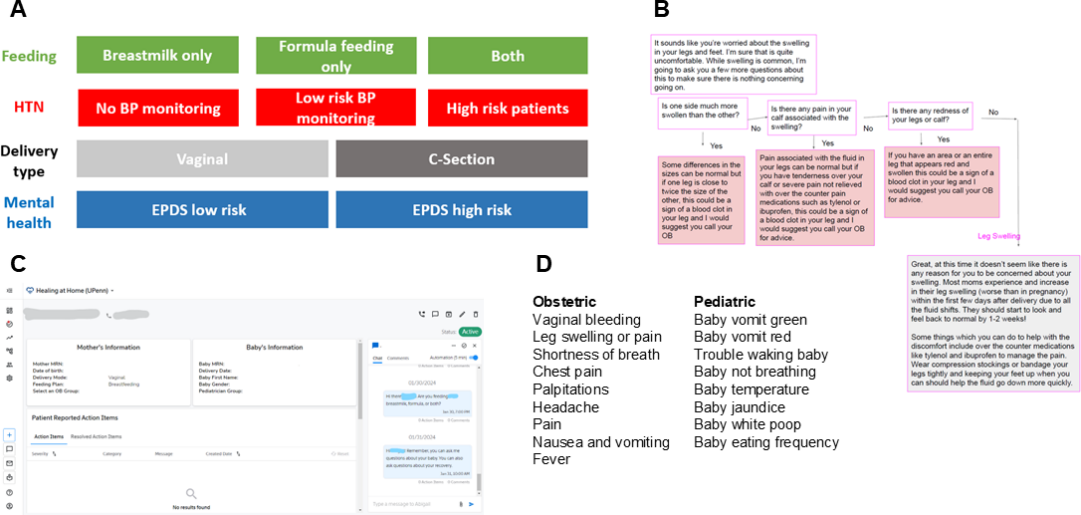
Layering of patient clinical characteristics (1A), example of clinical algorithm with symptom triage for lower extremity edema (1B), Memora Health patient dashboard (1C), comprehensive list of clinical symptoms for which algorithms were developed (1D). BP: blood pressure; EPDS: Edinburgh Postnatal Depression Screen; HTN: hypertension.

We partnered with Memora Health to undertake a 4-step process to develop the conversational agent using NLP to interact with patients in a HIPAA (Health Insurance Portability and Accountability Act)-compliant manner. Unlike a basic chatbot which uses rigid decision trees to respond to people, this type of conversational agent leverages NLP to understand and interpret patient messages, providing appropriate responses, leading to a conversational experience. First, a frequently asked question bank was used to generate accurate mapping of questions to the appropriate responses. Second, surveys (standardized conversation templates designed to collect patient data) were created by patients’ clinical characteristics (ie, breastmilk vs formula fed, Figure 1A). Third, creation of anticipatory guidance specific to patient clinical characteristic was planned. Finally, algorithms for potentially acute clinical concerns were designed and layered onto the program. Throughout this process we incorporated personal touches into responses, such as patients or infants’ names and worked to develop a consistent and empathetic tone.

The frequently asked questions were generated from both patients (through our 90-patient preintervention pilot) and clinicians (obstetrics, neonatology, lactation, and social workers on our mother-baby unit). Clinicians were encouraged to “think like a patient” and ask questions they had either received or conceived as important. An example question might be whether nipple pain with feeding is normal. Topics from both patients and providers were categorized (obstetrics, neonatology, or lactation), reviewed by our team for accurate clinical content and then made available in a frequently asked question bank.

Surveys, that is, structured questions designed to collect patient data, were used and incorporated into this program including validated clinical questionnaires such as the EPDS and net promoter score (NPS). The NPS is a customer satisfaction and loyalty metric used to measure the likelihood that customers will recommend a product, service, or experience to others. People are asked to provide a score from 0 to 10. Promoters are those who score a program 9 or 10, passives score of 7 or 8, and detractors 6 or less. The NPS is calculated by subtracting the percentage of promotors from the percentage of detractors. Scores of 50 or greater indicates exceptional loyalty. The NPS was collected from patients during week two of the program. We also developed multiple surveys with branching logic to dynamically respond to patients around topics such as infant feeding and the importance of attending scheduled appointments. Surveys were added to the program at scheduled times according to clinical needs.

Next, structured anticipatory guidance customized to patient characteristics (Figure 1) was generated by our clinical team such that patients with certain characteristics received appropriate educational materials at the right time (when they needed it and not before). Examples of customized anticipatory guidance include information on the volume of feeds by feeding method (breast vs formula).

Finally, we created a series of algorithms designed to address specific clinical scenarios outside of the conversational agent. For example, when asking about lower extremity edema, it cannot be assumed that this is normal swelling post partum, so triage regarding possible signs of venous thromboembolism is essential for providing safety (Figure 1). We also developed a “latch” algorithm, which was designed to address some common concerns in early lactation and difficulty with breastfeeding.

All content, including ad hoc content, surveys, anticipatory guidance, and clinical algorithms, was reviewed not only by our internal clinical leads but also by an expert clinical panel. This external panel, composed of a group of clinician leaders at our institution uninvolved with the design or development, provided us with insight and perspective on the content, our approach, and identification of perceived patient risks in development.

While we aimed to minimize unnecessary escalation, we erred on the side of caution with standard language and responses. For example, if the conversational agent is unable to answer a patient’s concern about their infant, they would receive the following response: “It sounds like this is something that infant name’s doctor can help with. Please call their office at 555-555-555.” We also instructed patients to use the phrase “TEXT ME” to indicate that their concern was not addressed, alerting the team to review the conversation and intervene. This was primarily accomplished through messaging patients directly in the Memora Health dashboard. Best practices when interacting with the conversational agent, such as using single-sentence questions and rephrasing questions to improve responses, were shared with the patients through flyers and a short educational video at the time of the program start.

### Conversational Agent Testing

Testing of the conversational agent required multiple phases, including internal and external testing. First, we tested this program internally by asking our own team members to ask questions that they would imagine patients might ask, attempting to cover a wide range of common clinical scenarios. Suggested scenarios included concerns around the color of infant stools, pain management, etc. Accuracy of the responses was also improved through a rapid-fire test using Mechanical Turk as described in detail by Lin et al [19]. Once these tests had been completed, we tested the program with providers external to our team and a small set of patients.

First, we recruited 23 providers from obstetrics, neonatology, nursing, and lactation who had not been involved in the design of the program to use the conversational agent as if they were a patient (they were assigned a patient characteristic such as feeding method for testing). In the second phase, we recruited 37 patients from the HUP postpartum unit to use the conversational agent in their own recovery. These patients were selected to be representative of our population with examples of demographics including race, parity, marital status, insurance, and age. During this initial patient testing phase, monitoring of the platform occurred once daily at a minimum by our clinical team.

### Chatbot Enrollment and Clinical Monitoring

Clinical criteria for patient participation in Healing at Home was determined by our clinical team and expert panel at the start of this program. Program participants were patients who were planned for discharge around 24 hours of after birth who had an uncomplicated vaginal delivery at term of a singleton infant; full exclusion criteria are outlined in Textbox 1. These clinical criteria were selected as clinicians felt most comfortable with a new technology being used by a group of patients less likely to experience postpartum complications. The data presented here regarding patient engagement includes 290 patients who met these clinical characteristics. Upon discharge, patients were enrolled in the texting platform by the postpartum nurse and verbally consented. Our first nontest patient was enrolled March 6, 2020. Once the program was live, we took a data-driven approach to improve the patient experience and the conversational agent itself. For example, when we discovered that many patients were asking about their infant’s umbilical cord care during the first week, we programmed a message to be proactively sent at that time.

Textbox 1. **Clinical exclusion criteria to enrollment in the “Healing at Home” program (planned discharge around 24 h post birth).**

Maternal exclusion criteria:

- Age<18
- Cesarean delivery
- Gestational age<37 weeks and 0 days
- Multiple gestation
- Blood loss>1000cc
- 3rd or 4th degree perineal laceration
- Preexisting diabetes mellitus or gestational diabetes on medication
- Preeclampsia with severe features
- Chronic hypertension on medication

Infant exclusion criteria:

- Intensive care nursery admission
- Birth weight<2500 g
- Direct antiglobulin test positive
- 24-hour glucose<50 mg/dL
- 24-hour bilirubin>6 mg/dL
- No void at 24 hours of life
- Elevated sepsis risk score (≥0.7)
- Weight loss >7% at 24 hours of life
- <6 feeds in first 24 hours

Other exclusion criteria:

- No access to texting
- Non-English speaking primary language
- Adoption case
- Department of Human Services involvement
- Patient opt out
- Provider opt out
- Latch score ≤6

While interactions are designed to be automated, it was assumed that unanswered questions or clinical concerns would occur. Clinical teams were assigned to respond to these escalations which were assigned an acuity level by our team as appropriate. Some alerts were received through email, while more critical alerts were sent by SMS text messages if deemed to be emergent. While patients interacted with the conversational agent by text message, monitoring of the program occurred through the Memora Health dashboard, where patients could be viewed, chat history seen, and patient characteristics could be edited as appropriate (for example, changing the feeding method from breast milk to formula). On the dashboard, clinicians could directly message with patients in addition to traditional methods of patient contact by phone (Figure 1C).

### Collection of Patient Engagement Data

A complete summary of clinical outcomes regarding users of this platform is beyond the scope of this paper, but we present demographic and engagement data from the first 6 months of users. This study was approved as a Quality Improvement Project by the University of Pennsylvania Institutional Review Board. Demographic data including age, parity, race, feeding method and insurance were collected from our electronic medical record. Patient engagement metrics and chatbot accuracy were extracted from individual patient text messages reviewed manually by our investigators. Classical descriptive statistics were generated using mean and SD for continuous variables and frequency and percentage for categorical variables. To measure the differences between demographically different groups, the chi-square test was used for categorical variables, and *t* test and ANOVA for continuous variables. Spearman rank correlations were used to describe relationships between continuous variables. All statistical analysis was performed using Stata (StataCorp).

Engagement with the chatbot was measured by the number of total texts, number of questions asked, and survey response rates. Questions were classified by content category (maternal, baby, lactation, and social work) and by whether the question was prompted or unprompted. Prompted questions were defined as questions related to a previous message (ie, “Remember you can ask me questions about your health or baby”) or directly following another interaction. Messages that were unrelated or temporally distant (>3 h since last message) were defined as unprompted. Binary data (asked vs did not ask question) and the total number of questions were recorded. Reworded questions did not count towards the total number of questions asked. Interactions between patients by a pleasantry (emoji, “ok,” “thanks!”) were recorded in a binary fashion. Patient satisfaction was collected through the NPS. Chatbot accuracy was measured by the percentage of correct answers per patient, excluding ignored interactions and no content situations. No qualitative interviews were conducted.

### Ethical Considerations

This study was approved as a Quality Improvement Project by the University of Pennsylvania Institutional Review Board.

## Results

A total of 290 patients used our chatbot over the first 6 months of use from March to August 2020. The average patient age was 28.8 (SD 5.47) years, 112 out of 290 (38.6%) patients were first time parents, 134 (46%) had private insurance, and 163 (56%) were Black (Table 1). This distribution is representative of the population at our large urban academic medical center. Of these 290 patients, 286 (98.6%) responded to the platform at least once, with 271 (93.4%) completing at least one survey, 151 (52%) asking a question (prompted or unprompted), and 162 (55.9%) interacting by a pleasantry. All patients were sent the EPDS at least 3 times over 6 weeks with 128 (44%) patients completing at least one EPDS. In addition, 93 (32%) of patients completed an NPS with an overall NPS score of 34.

**Table 1.** Demographic characteristics of first 6 months of users (N=290).

Demographic characteristics		n (%)
Age		
	<20	14 (4.8)
	20‐29	139 (48.1)
	30‐39	130 (44.7)
	≥40	7 (2.4)
Parity		
	0	112 (38.6)
	1	94 (32.4)
	2	51 (17.6)
	≥3	33 (11.7)
Race and ethnicity		
	Asian	13 (4.5)
	Black	163 (56)
	East Indian	3 (1)
	Hispanic Latino/Black	3 (1)
	Hispanic Latino/White	10 (3.4)
	Other	11 (3.8)
	Patient declined	2 (0.7)
	Unknown	2 (0.7)
	White	83 (28.6)
Insurance		
	Private	134 (46)
	Medicaid	156 (54)
Feeding type		
	Breast	194 (66.9)
	Formula	43 (14.8)
	Both	53 (18.3)

**Figure 2.**
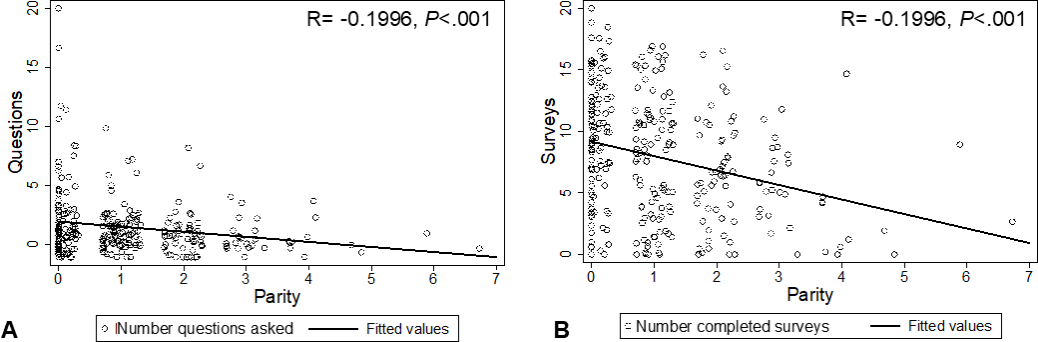
Parity versus number of questions asked (2A) and completed surveys (2B). Dots represent individual patients and were jittered to minimize overplotting.

Black patients were statistically more likely to promote the program (score 9 or 10 on a scale of 0‐10; *P*=.047) with an NPS score of 53 compared a NPS score of 18 for White patients. Engagement through survey completion and questions asked is shown in Table 2. White patients completed more surveys than Black patients (10.64 vs 6.64; *P*<.001), but there was no significant difference in the number of questions asked. Patients with private insurance completed more surveys than those with Medicaid (9.83 vs 6.43; *P*<.001), however, again no difference in questions asked. Patients feeding their infant breastmilk were more likely to ask questions (8.92 vs 4.65; *P*<.001) and complete surveys (10 vs 4; *P*<.001). There were a total of 32 “super users” (patients who asked more than 4 questions) of which 25 (78%) were non-White, with 19 (59.3%) of these “super users” being Black and exclusively breastfeeding (although only 87 out of the 290 patients in this cohort (30%) were both Black and exclusively breastfeeding; see Figure 2A). Patients with lower parity, that is, patients who had experienced their first birth, asked more questions (*P*<.001) and completed more surveys (*P*<.001) than patients who had already birthed 1 or more children. Each unit increase in parity decreased the total number of questions by 0.36 (Figure 2B).

**Table 2.** Engagement data by patient demographics.

Demographic		Black race	White race	Other race	*P* value	Private insurance	Medicaid insurance	*P* value	Breastmilk only	Formula only	Both	*P* value
Median total questions (IQR)		0 (0‐2)	1 (0‐2)	1 (0‐2)	.64	1 (0‐2)	0 (0‐2)	.26	1 (0‐2)	0 (0‐0)	1 (0‐2)	<.001
Total questions^a^, n (%)					.89			.26				<.001
0		82 (50)	39 (47)	18 (41)		60 (45)	79 (51)		83 (43)	33 (77)	23 (43)	
1		37 (23)	20 (24)	11 (25)		32 (24)	36 (23)		46 (24)	6 (14)	16 (30)	
2		18 (11)	7 (8)	6 (14)		12 (9)	19 (12)		42 (10)	1 (2)	10 (19)	
3+		26 (16)	17 (20)	9 (20)		30 (22)	22 (14)		45 (23)	3 (7)	4 (8)	
Total questions^b^, n (%)					.31			.94				.03
<4		141 (87)	76 (92)	41 (93)		119 (89)	139 (89)		166 (86)	42 (98)	50 (94)	
4+		22 (13)	7 (8)	3 (7)		15 (11)	17 (11)		28 (14)	1 (2)	3 (6)	
Completed surveys, median (IQR)		6 (3-10)	12 (7-15)	8 (4-12)	<.001	10 (6-14)	6 (3-10)	<.001	10 (5-13)	4 (1-7)	7 (4-9)	<.001

a Total number of patients with 0,1,2, and 3+ questions.

b Total number of patients with <4 or 4+ questions.

A total of 422 questions were asked by patients, 177 (42%) were prompted and 244 (58%) were unprompted. In addition, 211 (50%) questions of patient questions were related to infant concerns, 135 (32%) to maternal health, 72 (17%) to lactation concerns, and 4 (1%) to social work concerns. Approximately, 325 (73%) of all patient questions could be answered by the conversational agent with an overall chatbot accuracy of 77% (correctly answered questions/correctly answered plus incorrectly answered questions) with no difference in accuracy by parity, race, or insurance status. The additional 97 (27%) questions were not answered as they occurred concurrently with a survey (58/97) or had no developed content (39/97), these are excluded from the overall accuracy rate reported here. As this was a fluid development process, responses were created to questions that were missing content for future users.

## Discussion

Here we report on the development of a comprehensive postpartum conversational agent that leverages NLP to support patients during the “fourth trimester.” Satisfaction from patients using this texting program was the highest among Black patients with high rates of engagement by all users regardless of race. The maternal health crisis is real in the United States and impacts Black patients at significantly higher rates [5,20]. Contrary to the current design of prenatal care where emphasis is placed on the pregnant patient and not the postpartum patient, we aimed to design a scalable approach to support patients during the fourth trimester by SMS text messages using augmented intelligence and NLP through a novel postpartum conversational agent. Its holistic rather than problem-based design gives this technology the potential for scalability beyond what previous models or interventions have been able to achieve. We have shown here that patient engagement is high (>98% interaction rate and >93% survey completion rate) and that patient satisfaction in Black patients is high, with Black patients were more likely to promote this program than White patients (*P*=.047). As we look to solutions for the maternal health crisis, we must keep a critical eye on the impact that racism has on health and find solutions that specifically target these disproportionately impacted populations.

A confounding aspect to the engagement data presented here is the time during which we collected data: March-August 2020. Our go-live date for the program coincided very closely to the start of the shut-down related to the COVID-19 pandemic with local restrictions going into effect in the second week of patient use with this platform. The influence of this may have had a significant impact on patients’ experience with this platform and health care in general, especially in this cohort of patients who all completed the program by the end of 2020. Yet, we have continued to use this technology at our institution and will be able to determine whether and how moving out of the global pandemic impacts user engagement and patient satisfaction. Given the iterative nature of development, additional limitations include that significant improvements that were made over time (NLP improvement, mapping improvement) may not be reflected here (such as accuracy). Engagement by feeding method is confounded by race, with Black patients less likely to be exclusively breastfeeding, and NPS results are confounded by low response rates (<30%). An additional limitation of our engagement data presented here is that no qualitative interview of patients was performed. Without qualitative feedback from patients it is hard to draw any conclusions about the reason for NPS score disparity by race.

There are several outstanding questions that we have and plan to address in future work with this technology. First, we plan to report on the clinical outcomes on a larger cohort of patients with a specific focus on health care use and postpartum health goals such as visit attendance rates, ED and readmission rates as well as breastfeeding and contraception acceptance. In terms of health care use, we hope to gather data on number of phone calls to the office as well as amount of time per patient needed to manage concerns. In addition to these clinical and health care use outcomes, a key component to successful and broad implementation of such a program is intentional learning from the patients and providers who use this program. This allows for continued improvements and iterations on the program. We hope that future qualitative work with both providers and patients will help to elucidate barriers and facilitators to such a program. Within the framework of our layered program design, we have purposefully designed for flexibility in who, for instance, is asked to manage alerts or what content to include in the program to fit different hospital systems and teams.

**Figure 3.**
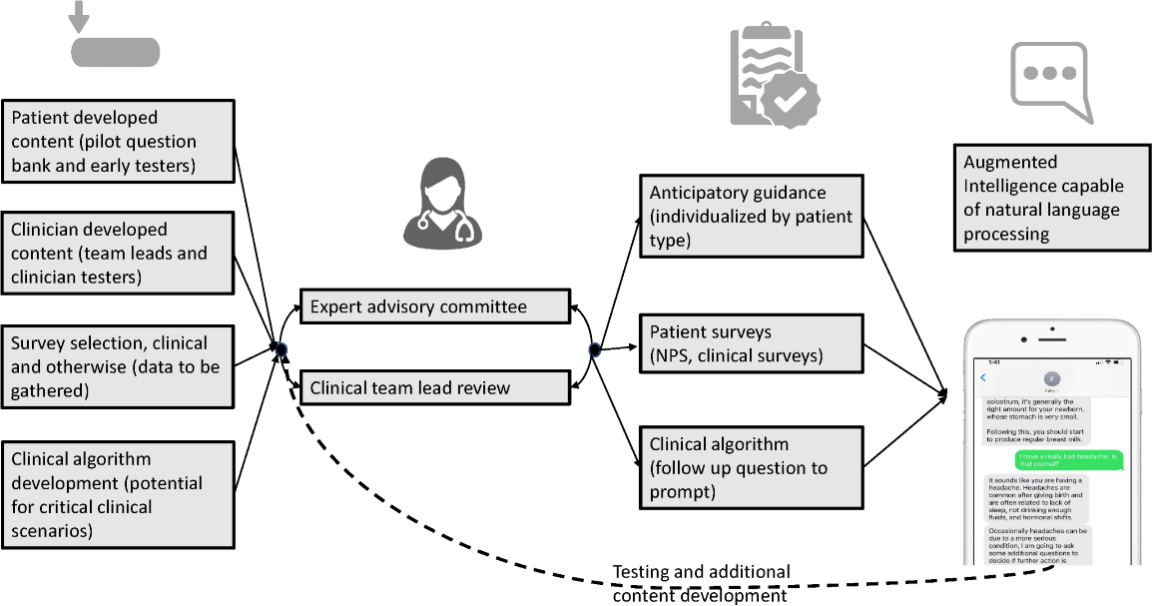
Conceptual framework for development of health care conversational agent. NPS: net promotor score.

We continue to use and expand upon this program at our own institution with to date over 1800 patients using this postpartum SMS text message–based support. Beyond our own application, we very much hope that the framework for the development of a comprehensive health care conversational agent (Figure 3) can help other clinical teams in their development, regardless of the specific clinical need addressed.

## Abbreviations

ACOG: American College of Obstetricians and Gynecologists
EPDS: Edinburgh Postnatal Depression Screen
HIPAA: Health Insurance Portability and Accountability Act
HUP: Hospital of the University of Pennsylvania
NLP: natural language processing
NPS: net promotor score
